# Tumour morphology of early-onset breast cancers predicts breast cancer risk for first-degree relatives: the Australian Breast Cancer Family Registry

**DOI:** 10.1186/bcr3248

**Published:** 2012-08-28

**Authors:** Gillian S Dite, Enes Makalic, Daniel F Schmidt, Graham G Giles, John L Hopper, Melissa C Southey

**Affiliations:** 1 Centre for Molecular, Environmental, Genetic and Analytic Epidemiology, The University of Melbourne, Melbourne, VIC, Australia; 2 Cancer Epidemiology Centre, Cancer Council Victoria, Carlton, VIC, Australia; 3 Department of Pathology, The University of Melbourne, Melbourne, VIC, Australia

## Abstract

**Introduction:**

We hypothesised that breast cancer risk for relatives of women with early-onset breast cancer could be predicted by tumour morphological features.

**Methods:**

We studied female first-degree relatives of a population-based sample of 452 index cases with a first primary invasive breast cancer diagnosed before the age of 40 years. For the index cases, a standardised tumour morphology review had been conducted for all; estrogen (ER) and progesterone receptor (PR) status was available for 401 (89%), and 77 (17%) had a high-risk mutation in a breast cancer susceptibility gene or methylation of the *BRCA1 *promoter region in peripheral blood DNA. We calculated standardised incidence ratios (SIR) by comparing the number of mothers and sisters with breast cancer with the number expected based on Australian incidence rates specific for age and year of birth.

**Results:**

Using Cox proportional hazards modelling, absence of extensive sclerosis, extensive intraductal carcinoma, absence of acinar and glandular growth patterns, and the presence of trabecular and lobular growth patterns were independent predictors with between a 1.8- and 3.1-fold increased risk for relatives (all *P *<0.02). Excluding index cases with known genetic predisposition or *BRCA1 *promoter methylation, absence of extensive sclerosis, circumscribed growth, extensive intraductal carcinoma and lobular growth pattern were independent predictors with between a 2.0- and 3.3-fold increased risk for relatives (all *P *<0.02). Relatives of the 128 (34%) index cases with none of these four features were at population risk (SIR = 1.03, 95% CI = 0.57 to 1.85) while relatives of the 37 (10%) index cases with two or more features were at high risk (SIR = 5.18, 95% CI = 3.22 to 8.33).

**Conclusions:**

This wide variation in risks for relatives based on tumour characteristics could be of clinical value, help discover new breast cancer susceptibility genes and be an advance on the current clinical practice of using ER and PR as pathology-based predictors of familial and possibly genetic risks.

## Introduction

While information about breast tumour morphology is used to make important decisions about treatment, it can also be important in assessing breast cancer risk for an affected woman's relatives. In this paper, we look at the extent to which familial risk of breast cancer depends on tumour morphology, an issue of both clinical and aetiological significance.

Female relatives of women with breast cancer are at increased risk of the disease, and it is well-established that the magnitude of their risk depends upon the closeness of their relationship to their affected relatives and the number and age at diagnosis of their affected relatives [1-6]. The risk of breast cancer is substantially elevated for women with an affected first-degree relative carrying a high-risk mutation in *BRCA1 *or *BRCA2 *[4-6] or a mutation in *ATM *[7] or *TP53 *[8-10], but high-risk mutations in these susceptibility genes are rare and explain only approximately 25% of the familial risk of breast cancer [11].

We recently showed that two breast tumour morphology features, trabecular growth pattern and high mitotic index, were sufficient to identify almost all *BRCA1 *mutation carriers in our population-based sample of breast cancer cases diagnosed before the age of 40 years [12]. We also found that having five or more of nine pre-specified tumour morphology features defined a morphological profile that contained 27 (93%) of the 29 known *BRCA1 *mutation carriers [12], and of the 52 with this morphological profile who were not found to carry a germline *BRCA1 *mutation, 16 (31%) were found to have methylation of the *BRCA1 *promoter region in DNA from peripheral blood [13].

Given the importance of tumour morphology features to inform treatment choices and to identify *BRCA1 *mutation carriers [12] or *BRCA1 *promoter methylation [13], we are now interested in identifying tumour morphology features that predict familial risk of early-onset breast cancer.

## Methods

### Australian Breast Cancer Family Registry

This study used data and materials from the Australian Breast Cancer Family Registry (ABCFR), which includes a population-based case-control-family study that has been described in detail elsewhere [3,14-16]. Briefly, between 1992 and 1998, index cases were identified using state population-based cancer registries as adult women living in the metropolitan areas of Melbourne and Sydney, who were aged less than 60 years when diagnosed with a histologically confirmed first primary invasive breast cancer. Index cases and participating relatives completed an interviewer-administered risk factor questionnaire and a family history questionnaire that asked for details of any cancer history for themselves and for their first-degree and second-degree relatives [3].

Missing family history data were imputed using a previously developed protocol for the following data items: date of birth, age, vital status, date of death, age at death, date at breast cancer diagnosis and age at breast cancer diagnosis [3,6]. Verification of reported cancers through cancer registries, medical records and death certificates was obtained where possible [3].

Approval for the study was obtained from the Human Research Ethics Committees of the University of Melbourne and the Cancer Councils of Victoria and New South Wales. All participants provided written informed consent prior to participation in the ABCFR.

### Tumour morphology review

Archival tumour blocks were sought for all index cases under the age of 40 years at diagnosis [12], and a validated tumour morphology review was completed by pathologists blinded to the mutation status of the index case [17,18]. Briefly, the tumours were typed into primary pattern and secondary pattern using the World Health Organisation breast carcinoma classification with minor modifications as described by Page *et al*. [19]. Tumour grade was scored using the modified system of Bloom and Richardson by assessing mitotic rate, tubular differentiation and nuclear pleomorphism [19] as applied in Southey *et al*. [12]. Sclerosis was defined as a central area of fibrosis composed of fibroblasts and/or collagen that is devoid of tumour cells, and labelled extensive if it comprised more than 20% of the tumour volume.

Estrogen receptor (ER) and progesterone receptor (PR) status was obtained either from immunohistochemical testing of tumour tissue [20] or from histopathology reports held by the cancer registry or a diagnostic laboratory [21].

From the tumour morphology review, the following features were extracted as either absent or present:

Number of mitoses >50 (per 10 high powered fields)

Nuclear grade - malignant (scored as bland, intermediate, malignant)

Tubule formation <10 (scored as >75, 10 to 75, <10)

Syncytical pattern ≥75% (scored as no, yes)

Circumscribed (scored as no, yes)

Pushing margins >50% (scored as no, yes)

Lymphocytic Infiltrate I - diffuse (scored as absent/minimal, border, diffuse)

Lymphocytic infiltrate II - intense (scored as absent/minimal, moderate, intense)

Glandular growth pattern (primary growth pattern)

Lobular growth pattern (primary or secondary growth pattern)

Trabecular growth pattern (primary or secondary growth pattern)

Tubular growth pattern (primary or secondary growth pattern)

Acinar growth pattern (secondary growth pattern)

Sclerosis - extensive (scored as minimal, extensive)

Extensive intraductal carcinoma - present (scored as absent, present, uncertain)

Necrosis - present (scored as absent, present, uncertain)

Apoptosis - moderate or intense (scored as absent/minimal, moderate, intense)

Lymphatic invasion - present (scored as absent, present, uncertain)

Note: growth patterns were scored as glandular (primary only), lobular, trabecular, tubular and acinar (secondary only).

### Mutation testing for high-risk breast cancer genes

Testing for high-risk mutations in breast cancer predisposition genes was conducted for all index cases under the age of 40 years at diagnosis and from whom a blood sample had been obtained. Full details of the *BRCA1 *and *BRCA2 *mutation testing have been reported elsewhere and included: sequencing, protein truncation testing, two-dimensional gel electrophoresis, screening for large genomic alterations, testing for Ashkenazi founder mutations and testing for duplication of exon 13 of *BRCA1 *[3,12]. *ATM *was tested for the c.7271 T>G mutation [22-25] and testing for mutations in *TP53 *was performed by Sanger sequencing and specific screening for larger gene rearrangements [10].

### *BRCA1 *methylation

Testing for methylation of the promoter region of *BRCA1 *was conducted for all index cases under the age of 40 years at diagnosis and for whom a pathology review had been conducted [13].

### Relatives

For the present study, mothers and sisters were included if their index case was under the age of 40 years when diagnosed with a histologically confirmed first primary invasive breast cancer and had a pathology review completed. For each relative included in the analyses, the following data were extracted: relationship to the index case; date of birth; vital status; age at interview or death; breast cancer status; and age at diagnosis, if affected.

### Statistical methods

Australian population-based female breast cancer incidence rates, specific for age and year of birth (both in five-year groupings), were obtained for 1983 to 2001 and extrapolated to earlier years as described previously [6]. The standardised incidence ratio (SIR) was estimated by comparing the observed number of mothers and sisters with breast cancer with the number expected from applying Australian population-based female breast cancer incidence rates specific for age and year of birth (both in five-year groupings). The ratio of SIRs and 95% confidence intervals (CI) were calculated using the method described by Breslow and Day [[26], pages 94-95]. Estimates of hazard ratios (HR) were obtained from Cox proportional hazards models and robust estimates of standard errors were obtained to account for clustering within families. We used the likelihood ratio test to identify best fitting models by forward selection and confirmed by backwards elimination, with a nominal *P*-value of 0.05 as a threshold, and to test the null hypothesis that there is no association between a set of predictive features and risks for relatives.

Multivariate linear regression models were also used for estimating and testing associations between the measured pathology features and risk for relatives. These models were fitted using Bayesian regularised regression with the Least Absolute Shrinkage and Selection Operator (LASSO) penalty function [27,28]. The outcome variable was the difference between expected and observed SIRs, while the pathology features were used as binary explanatory variables. To estimate the logistic regression parameters, we generated 10^5 ^samples from the posterior distribution of the parameters given the data, discarded the first 2 × 10^4 ^samples as *burnin*, and *accepted *every fifth posterior sample, thus reducing autocorrelation within the chain.

Because many of the pathology features were highly correlated, Bayesian LASSO regression and the Cox proportional hazards model were used in tandem to select features to be included in the multivariate model.

Statistical analyses were performed with Stata version 11, StataCorp LP, College Station, Texas, USA [29] and MATLAB^® ^version 7.13, MathWorks, Natick, Massachusetts, USA [30]. All statistical tests were two-sided and *P*-values <0.05 were considered nominally statistically significant.

## Results

A total of 856 index cases diagnosed with a first primary invasive breast cancer before the age of 40 years was recruited, of whom 452 had a tumour morphology review using archived tissue. Of these, 77 (17%) had either a high-risk mutation in a breast cancer susceptibility gene or methylation of the promoter region of *BRCA1 *in peripheral blood DNA (31 *BRCA1*, 16 *BRCA2*, 1 *ATM*, 4 *TP53*, 25 *BRCA1 *promoter region methylation). The remaining 375 (83%) constituted the subgroup for further analyses. Table 1 shows the distribution of each tumour morphology feature for all the 452 index cases and for the 375 index cases in the subgroup. ER and PR status was available for 401 (89%) of all index cases and for 329 (89%) of the subgroup of index cases.

**Table 1:** Number and percentage of index cases with each of the tumour morphology features

Feature	All index cases		Subgroup of index cases	
	N = 452^†^	%	N = 375^‡^	%
Number of mitoses >50	71	15.7	33	8.8
Nuclear grade - malignant	386	85.4	316	84.3
Tubule formation <10	344	76.1	283	75.5
Syncytial pattern ≥75%	36	8.0	21	5.6
Circumscribed	93	20.6	56	14.9
Pushing margins	16	3.5	11	2.9
Lymphocytic infiltrate I - diffuse	162	35.8	136	36.3
Lymphocytic infiltrate II - intense	96	21.2	69	18.4
Glandular growth pattern	307	67.9	272	75.5
Lobular growth pattern	162	35.8	147	39.2
Trabecular growth pattern	109	24.1	65	17.3
Tubular growth pattern	74	16.4	64	17.1
Acinar growth pattern	87	19.3	60	16.0
Sclerosis - extensive	420	92.9	356	94.9
Extensive intraductal carcinoma	70	15.5	63	16.8
Necrosis	173	38.3	126	33.6
Apoptosis - moderate or intense	362	80.1	294	78.4
Lymphatic invasion	141	31.2	117	31.2
ER positive	226	56.4	201	61.1
PR positive	258	64.3	219	66.6

† ER and PR status was available for 401 index cases.

‡ ER and PR status was available for 329 index cases.

There were 1,041 relatives (452 mothers and 589 sisters) of the index cases, an average of 2.3 relatives per index case, with a total of 11,786 person-years of observation (6,340 person-years for mothers and 5,446 person-years for sisters). Data were imputed for relatives with missing data as follows: seven dates of birth; six vital status; four dates and ages at death; and one date and age at breast cancer diagnosis.

Overall, 106 breast cancers were observed for the mothers and sisters of the index cases while 36.56 were expected from population incidence rates. The ratio of observed to expected, the SIR, was 2.90 with a 95% CI from 2.40 to 3.51.

Table 2 shows, for the presence and absence of each of the pathology features, the number of observed and expected cases of breast cancer, the SIR and 95% CI. Table 2 also shows the ratio of SIRs and corresponding 95% CI and the *P*-values as a test of the difference between the SIRs for the presence and absence of each pathology feature. The test of the null hypothesis of no association with these variables and risks for relatives was rejected (Χ_18_^2 ^= 106; *P *= 10^-14^). After fitting the five significant morphology features, there was still significant variation in risks for relatives across the remaining 13 features (Χ_13_^2 ^= 30; *P *= 0.004).

**Table 2:** SIRs for mothers and sisters by tumour morphology features of the 452 index cases: the Australian Breast Cancer Family Registry.

Feature		Observed	Expeceted	SIR	(95% CI)	Ratio of SIRs	(95% CI)	*P*
**Number of mitoses >50 **	Absent	81	31.70	2.56	(2.06 to 3.18)	2.01	(1.23 to 3.19)	0.002
	Present	25	4.86	5.14	(3.47 to 7.61)			
**Nuclear grade - malignant**	Absent	13	5.32	2.44	(1.42 to 4.21)	1.22	(0.68 to 2.37)	0.5
	Present	93	31.24	2.98	(2.43 to 3.65)			
**Tubule formation <10**	Absent	21	9.18	2.29	(1.49 to 3.51)	1.36	(0.83 to 2.30)	0.2
	Present	85	27.38	3.10	(2.51 to 3.84)			
**Syncytial pattern ≥75%**	Absent	92	34.20	2.69	(2.19 to 3.30)	2.21	(1.16 to 3.89)	0.01
	Present	14	2.36	5.93	(3.51 to 10.01)			
**Circumscribed**	Absent	70	29.51	2.37	(1.88 to 3.00)	2.15	(1.40 to 3.26)	<0.001
	Present	36	7.05	5.11	(3.68 to 7.08)			
**Pushing margins**	Absent	96	35.40	2.71	(2.22 to 3.31)	3.15	(1.46 to 6.05)	0.001
	Present	10	1.17	8.58	(4.62 to 15.95)			
**Lymphocytic infiltrate I - diffuse**	Absent	58	23.20	2.50	(1.93 to 3.23)	1.44	(0.96 to 2.14)	0.06
	Present	48	13.36	3.59	(2.71 to 4.77)			
**Lymphocytic infiltrate II-intense**	Absent	73	29.83	2.45	(1.95 to 3.08)	2.00	(1.29 to 3.06)	0.001
	Present	33	6.73	4.90	(3.49 to 6.90)			
**Glandular growth pattern**	Absent	47	10.80	4.35	(3.27 to 5.79)	0.53	(0.35 to 0.79)	0.001
	Present	59	25.76	2.29	(1.77 to 2.96)			
**Lobular growth pattern**	Absent	62	22.80	2.72	(2.12 to 4.89)	1.18	(0.78 to 1.76)	0.4
	Present	44	13.76	3.20	(2.38 to 4.30)			
**Trabecular growth pattern**	Absent	70	28.65	2.44	(1.93 to 3.09)	1.86	(1.21 to 2.82)	0.002
	Present	36	7.91	4.55	(3.28 to 6.31)			
**Tubular growth pattern**	Absent	91	30.34	3.00	(2.44 to 3.68)	0.80	(0.43 to 1.39)	0.4
	Present	15	6.23	2.41	(1.45 to 4.00)			
**Acinar growth pattern**	Absent	90	30.03	3.00	(2.44 to 3.68)	0.82	(0.45 to 1.40)	0.5
	Present	16	6.53	2.45	(1.51 to 4.00)			
**Sclerosis - extensive**	Absent	20	2.36	8.46	(5.46 to 13.11)	0.30	(0.18 to 0.51)	<0.001
	Present	86	34.20	2.52	(2.04 to 3.11)			
**Extensive intraductal carcinoma**	Absent	81	30.74	2.34	(2.12 to 3.28)	1.63	(1.00 to 2.58)	0.03
	Present	25	5.82	4.30	(2.90 to 6.36)			
**Necrosis**	Absent	64	23.93	2.67	(2.09 to 3.42)	1.24	(0.82 to 1.86)	0.3
	Present	42	13.63	3.33	(2.46 to 4.50)			
**Apoptosis - moderate or intense**	Absent	16	7.30	2.19	(1.34 to 3.58)	1.40	(0.82 to 1.86)	0.2
	Present	90	29.26	3.08	(2.50 to 3.78)			
**Lymphatic invasion**	Absent	75	24.96	3.01	(2.40 to 3.77)	0.89	(0.57 to 1.37)	0.6
	Present	31	11.60	2.67	(1.88 to 3.80)			
**ER positive**	Absent	48	13.16	3.65	(2.75 to 4.84)	0.63	(0.41 to 0.96)	0.02
	Present	46	20.16	2.28	(1.71 to 3.05)			
**PR positive**	Absent	42	11.32	3.71	(2.75 to 5.02)	0.66	(0.43 to 1.01)	0.04
	Present	54	22.03	2.45	(1.88 to 3.20)			

Breast cancer risks for female first-degree relatives were increased if the index cases' tumours: were circumscribed; had pushing margins >50%; had intense lymphocytic infiltrate II; had number of mitoses >50; had a trabecular growth pattern; had extensive intraductal carcinoma; had a syncytial pattern; did not have extensive sclerosis; did not have a glandular growth pattern; or were ER or PR negative (all *P *<0.04).

Table 3 shows the HR, 95% CI and *P*-values for the multivariate model. When all features were considered together, the risks for relatives could be described by six predictive features: absence of extensive sclerosis;; acinar growth pattern; trabecular growth pattern; glandular growth pattern; lobular growth pattern; and extensive intraductal carcinoma (all *P *<0.02). When these factors were included in the model, there was at best marginally significant evidence for associations with tubular growth pattern (HR = 0.51; 95% CI 0.28 to 0.93; *P *= 0.03) and tubule formation (HR = 1.64; 95% CI 0.97 to 2.78; *P *= 0.07). Neither ER status nor PR status provided additional (i.e. independent) information on breast cancer risk for relatives (both *P *>0.3).

**Table 3:** Multivariate modelling of hazard ratios for tumour morphology features of the index cases.

Feature	Hazard Ratio	(95% CI)	*P*
Sclerosis - extensive	0.36	(0.20 to 0.65)	0.001
Acinar growth pattern	0.42	(0.22 to 0.78)	0.006
Glandular growth pattern	0.40	(0.25 to 0.65)	<0.001
Extensive intraductal carcinoma	1.96	(1.28 to 3.00)	0.002
Circumscribed	1.85	(1.10 to 3.12)	0.02

### Subgroup analyses

When restricted to the subgroup of 375 index cases, there were 876 relatives (375 mothers and 501 sisters), an average of 2.3 relatives per index case, with a total of 9,954 person-years of observation (5,287 person-years for mothers and 4,667 person-years for sisters). Overall, 69 breast cancers were observed and 30.74 were expected based on population incidence rates; the SIR was 2.25 with a 95% CI from 1.77 to 2.84.

Table 4 shows SIR and 95% CI and the ratio of the SIRs for each of the pathology features. Breast cancer risks for relatives were increased if the tumours: were circumscribed; had pushing margins; had extensive intraductal carcinoma; had glandular growth pattern; or did not have extensive sclerosis (all *P *<0.04). Neither ER status nor PR status provided independent information on breast cancer risk for relatives (both *P *>0.4). The test of the null hypothesis of no association with these variables and risks for relatives was rejected (Χ_18_^2 ^= 71.0; *P *= 10^-8^). Even after fitting the four significant morphology features there was still significant variation in risks for relatives across the remaining 13 features (Χ_14_^2 ^= 45.4; *P *= 0.0001).

**Table 4:** SIRs for mothers and sisters by morphology features of the subgroup of 375 index cases.

Feature		Observed	Expeceted	SIR	(95% CI)	Ratio of SIRs	(95% CI)	*P*
**Number of mitoses >50 **	Absent	60	28.50	2.11	(1.64 to 2.71)	1.91	(0.83 to 3.87)	0.07
	Present	9	2.24	4.02	(2.09 to 7.73)			
**Nuclear grade - malignant**	Absent	10	4.67	2.14	(1.15 to 3.98)	1.06	(0.54 to 2.32)	0.9
	Present	59	26.06	2.26	(1.75 to 2.92)			
**Tubule formation <10**	Absent	16	7.86	2.04	(1.25 to 3.32)	1.14	(0.64 to 2.13)	0.7
	Present	53	22.87	2.32	(1.77 to 3.03)			
**Syncytial pattern ≥75%**	Absent	64	29.42	2.18	(1.70 to 2.78)	1.75	(0.55 to 4.31)	0.2
	Present	5	1.31	3.81	(1.58 to 9.14)			
**Circumscribed**	Absent	51	26.29	1.94	(1.47 to 2.55)	2.09	(1.15 to 3.63)	0.01
	Present	18	4.45	4.04	(2.55 to 6.42)			
**Pushing margins**	Absent	66	29.98	2.10	(1.64 to 2.69)	3.76	(1.33 to 8.64)	0.002
	Present	6	0.76	7.90	(3.55 to 17.59)			
**Lymphocytic infiltrate I - diffuse**	Absent	38	19.42	1.96	(1.42 to 2.69)	1.40	(0.84 to 2.31)	0.2
	Present	31	11.32	2.74	(1.93 to 3.89)			
**Lymphocytic infiltrate II-intense**	Absent	53	25.89	2.05	(1.56 to 2.68)	1.61	(0.86 to 2.86)	0.1
	Present	16	4.85	3.30	(2.02 to 5.39)			
**Glandular growth pattern**	Absent	25	7.58	3.30	(2.23 to 4.88)	0.58	(0.34 to 0.98)	0.03
	Present	44	23.16	1.90	(1.41 to 2.55)			
**Lobular growth pattern**	Absent	36	18.34	1.96	(1.42 to 2.72)	1.36	(0.82 to 2.24)	0.2
	Present	33	12.40	2.66	(1.89 to 3.74)			
**Trabecular growth pattern**	Absent	54	25.90	2.09	(1.60 to 2.72)	1.49	(0.78 to 2.67)	0.2
	Present	15	4.84	3.10	(1.87 to 5.15)			
**Tubular growth pattern**	Absent	59	25.25	2.34	(1.81 to 3.02)	0.78	(0.36 to 1.54)	0.5
	Present	10	5.49	1.82	(0.98 to 3.39)			
**Acinar growth pattern**	Absent	62	26.10	2.37	(1.85 to 3.04)	0.65	(0.25 to 1.42)	0.3
	Present	7	4.56	1.54	(0.73 to 3.22)			
**Sclerosis - extensive**	Absent	8	1.45	5.52	(2.76 to 11.04)	0.38	(0.18 to 0.91)	0.01
	Present	61	29.29	2.08	(1.62 to 2.68)			
**Extensive intraductal carcinoma**	Absent	51	25.61	1.99	(1.51 to 2.62)	1.77	(0.97 to 3.06)	0.04
	Present	18	5.12	3.51	(2.21 to 5.58)			
**Necrosis**	Absent	48	21.35	2.25	(1.70 to 2.98)	0.99	(0.57 to 1.69)	1.0
	Present	21	9.39	2.24	(1.46 to 3.43)			
**Apoptosis - moderate or intense**	Absent	12	6.64	1.81	(1.03 to 3.18)	1.31	(0.69 to 2.68)	0.4
	Present	57	24.10	2.37	(1.83 to 3.07)			
**Lymphatic invasion**	Absent	53	21.10	2.51	(1.92 to 3.29)	0.76	(0.35 to 1.17)	0.1
	Present	16	9.64	1.66	(1.02 to 2.71)			
**ER positive**	Absent	25	10.00	2.50	(1.69 to 3.70)	0.65	(0.44 to 1.33)	0.3
	Present	34	17.85	1.91	(1.36 to 2.67)			
**PR positive**	Absent	26	9.10	2.86	(1.95 to 4.20)	0.70	(0.38 to 1.13)	0.1
	Present	35	18.78	1.86	(1.34 to 2.60)			

Table 5 shows the HR, 95% CI and *P*-values for the multivariate model. When all features were considered together, the pathology features identified by the Bayesian LASSO regression and the Cox proportional hazards models were: being circumscribed; having extensive intraductal carcinoma; having lobular growth pattern; and not having extensive sclerosis (all *P *<0.02). When these factors were included in the model, no other factors were even marginally associated.

**Table 5:** Multivariate modelling of hazard ratios for morphology features of the subgroup of 375 index cases.

Feature	Hazard Ratio	(95% CI)	*P*
Sclerosis-extensive	0.30	(0.13 to 0.70)	0.005
Circumscribed	2.91	(1.39 to 6.10)	0.01
Extensive intraductal carcinoma	2.21	(1.25 to 3.90)	0.01
Lobular growth pattern	2.01	(1.10 to 3.70)	0.02

**Figure 1:**
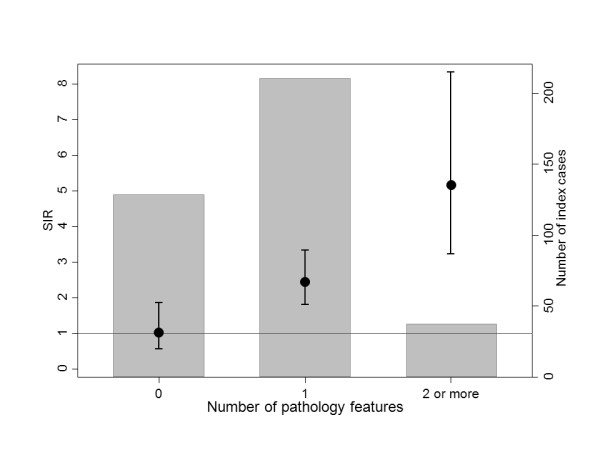
**SIRs (95% CIs) for mothers and sisters of 375 index cases by number of pathology features**. Grey areas represent the proportion of index cases with given number of features.

Figure 1 shows that, based on the model presented in Table 5, it would be predicted that women with breast cancer diagnosed before age 40 years who do not have a currently identifiable genetic risk, can be divided into three groups defined by the four pathology features. Relatives of the 128 (34%) index cases with none of these features are predicted to be at no increased risk (SIR = 1.03, 95% CI = 0.57 to 1.85) while relatives of the 37 (10%) cases who had two or more features were predicted to be at highest risk (SIR = 5.18, 95% CI = 3.22 to 8.33). The remaining 210 (56%) cases are predicted to be at about average increased risk (SIR = 2.45, 95% CI = 1.80 to 3.33).

**Figure 2:**
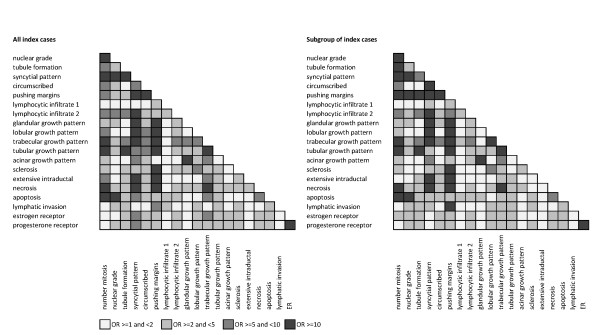
**Associations between pathology features for all index cases (left) and the subgroup of cases (right)**. Each row and column corresponds to a feature and the shading represents different levels of association, as measured by the odds ratios (ORs), as indicated. Odds ratios <1 were assigned the same levels as their reciprocals.

Figure 2 shows that some of the morphological and immunohistochemical features are strongly associated with one another, for both all index cases and for the subgroup of index cases; cf. Figure 2 of [12]. For example, notice that syncytial pattern and pushing margins are highly associated with each other and several other features for both groups.

## Discussion

For women with early-onset breast cancer who neither carry a mutation in a known breast cancer susceptibility gene nor have *BRCA1 *promoter region methylation in peripheral blood DNA, features of their tumour morphology predict wide variation in the risk of breast cancer for their first-degree female relatives, far more than could be attributed to chance. For example, female relatives of the approximately one-third of early-onset breast cancer cases who had tumours that lacked any morphological feature associated with familial risk were at no increased risk of breast cancer. Female relatives of the approximately one-sixth of early-onset breast cancer cases who had tumours that had two or more of the four features associated with familial risk were at a substantially increased risk of breast cancer. The latter finding suggests there are undiscovered strong familial risk factors for early-onset breast cancer. The former suggests there is a substantial proportion of early-onset breast cancer that does not have a familial, let alone genetic, cause.

This work has built on previous work by us and others that identified specific morphological features which predict cases with a germline *BRCA1 *mutation [12,31,32]. We recently extended this to show that those features also predict cases with methylation of the promoter region of *BRCA1 *in peripheral blood DNA [13]. Here, we have widened the scope of what we are predicting to risks for relatives and, at the same time, included all the other morphological features assessed by a standardised and validated pathology review [18]. We also assessed morphological predictors both including and excluding cases with a known genetic cause or methylation of the *BRCA1 *promoter region.

When first considering all index cases, we found that absence of extensive sclerosis, extensive intraductal carcinoma, and trabecular and lobular growth patterns and absence of acinar and glandular growth patterns were all independent predictors of breast cancer for their first-degree female relatives. When considered together, for each of these six features their presence was associated with an approximate doubling of risk for relatives. This means there is a substantial gradient of risk for relatives across a number of these features.

We have previously shown that the presence of trabecular growth pattern was the major predictor of *BRCA1 *mutation status, as well as of methylation of the *BRCA1 *promoter region [12,13]. When we excluded index cases with known genetic causes or methylation of the *BRCA1 *promoter region, absence of extensive sclerosis, circumscribed growth, extensive intraductal carcinoma and lobular growth pattern were each independent predictors of breast cancer risk for the first-degree female relatives of the remaining index cases. Note from Figure 2 that these factors are not highly associated with one another. Again, the strengths of prediction were each an approximate doubling or more of risk, and there was a substantial gradient in risk for the relatives associated with the number of these four features in the tumour of the index case. Therefore, this study has identified characteristics of breast cancers in young women, other than basal-like carcinoma (which is likely rare in our subgroup analyses due to exclusion of the index cases with either known *BRCA1 *germline mutations or methylation of the *BRCA1 *promoter region), that are associated with increased risks of breast cancer for first-degree relatives.

There are several strengths of our study. First, it is population-based, so inference can readily be made at least to the populations from which these index cases were sampled, if not more generally. Second, because the index cases were not selected on the basis of family history, this study gives insights across the whole spectrum of index cases. Third, we have used data from multiple pathologists conducting a standardised and validated tumour review and any systematic variation across the pathologists would mean that our study has under-estimated the predictive value of these morphology features.

The study also has some weaknesses. It is possible that, despite extensive testing, some index cases carrying mutations in *BRCA1 *and *BRCA2 *have not yet been identified, although this is likely to be, at most, a few percent [3,6]. There may be some false positive statistical results due to the vagaries of chance. The study has used only early-onset index cases, so we cannot infer that the same results will apply to index cases of later onset. Our index cases were predominantly Caucasian and we cannot infer that these results apply to other populations. Therefore, there is a need for replication using independent samples, and using samples of index cases with different ages at onset and different ethnic backgrounds. Finally, although we have identified a small number of features that independently predict risks for relatives in our data set, there was still substantial unexplained variation in risk. Therefore, larger and pooled studies might identify a bigger and better set of predictors. These might also clarify which factors are the better independent predictors, given that some features are highly associated with one another (see Figure 2).

While there are several studies of breast cancer in relatives as a function of the ER and PR tumour status of an affected woman (for example, [33]), few have studied histological features (for example, [34]). Several studies have shown that stratification of breast tumours by ER and PR status might be useful in partitioning breast cancer families into more homogeneous subsets [35], but we have found that these traditional immunohistochemical features are not as important as morphological features for predicting *BRCA1 *mutation carriers [12] or, as in this study, predicting breast cancer risks for relatives. Specific morphological types of breast cancer might be associated with familial cancers in general (or example, [36]).

Being able to quantify a woman's risk of breast cancer is important for genetic counselling, prevention and screening, as well as for aetiological research. Currently, risk estimates associated with having a family history are based on the age at diagnosis of cancer, not on the cancer's morphological features. We have shown here that, at least for first-degree relatives of women with early-onset breast cancer, this is likely leading to a misassignment of familial risks for about one-half of index cases, being too high for one-third and too low for one-sixth, by factors of two-fold or more. Assignment of women with a family history of breast cancer to different screening strategies based on their absolute risk could be substantially improved were the morphology features of the tumours of the relatives taken into account. The role of morphological features to predict *BRAC1 *mutation status has been established for some time [31]. For early-onset breast cancer, we showed that only two key features could better predict *BRCA1 *mutation carriers than family history and/or ER and PR tumour status [12]. Now we have shown that just four other morphological features predict familial risks after excluding the known breast susceptibility genes, and again better than ER and PR tumour status.

It is highly relevant to genetic research and counselling that one-third of early-onset breast cancers might not even have a heritable genetic basis, as evidenced by our observation that, for the group of index cases with none of the four predictive features, their relatives were not at increased risk. Most linkage studies and the recent genome-wide association studies have generally pooled all cases when trying to find predisposing genes. It is also of relevance to know if the type of breast cancers, as defined by the morphology features identified here, 'runs in the family', as this would greatly assist efforts at susceptibility gene discovery.

For the affected women, their germline status, as well as changes (both somatic genetic and epigenetic) to tumour DNA, might influence gene expression that manifests in nuances of cell growth and neoplastic tissue organisation that are able to be observed by microscope. For example, breast tumours arising in women with germline *BRCA1 *mutations and *TP53 *mutations have characteristic morphology and acquired genetic and epigenetic mutation profiles [37-39]. For unaffected relatives, any familial risk associated with the breast tumour morphology features of an affected relative must be related to similar genetic or epigenetic variants that increase cancer susceptibility. From this, it follows that DNA from affected women whose breast tumour morphology features are associated with the highest familial risks should be prioritised for comprehensive genetic and epigenetic screening as they provide the best prospect for the discovery of novel variants that are associated with risk.

On their own, the ER and PR status of the index cases were both associated with risk for relatives overall, with about a 50% increased risk if the tumour of the index case was negative for either of these immunohistochemical markers. After the exclusions, the associations were of the same magnitude and direction but no longer statistically significant. However, neither of these immunohistochemical markers were independent predictors of risks for relatives once the more strongly predictive morphological features were taken into account. That is, we present here an advance on using ER and PR alone to assess the role of pathology in predicting increased familial and possibly genetic risks.

## Conclusions

We have shown that relatively standard tumour morphology can help unravel the complex heterogeneity of breast cancers from the perspective of familial and genetic risk, at least in the setting of early-onset disease. There is a very wide variation in risks for relatives based on tumour characteristics and this could not only be of clinical value but might help discover new breast cancer susceptibility genes.

## Abbreviations

ABCFR: Australian Breast Cancer Family Registry; CI: confidence interval; ER: estrogen receptor; HR: hazard ratio; LASSO: least absolute shrinkage and selection operator; PR: progesterone receptor; SIR: standardised incidence ratio

## Competing interests

The authors declare that they have no competing interests.

## Authors' contributions

GSD led the writing of the manuscript, reviewed the literature, analysed and interpreted data, and also contributed to the study design and data collection. MCS established the ABCFR and its data collection, conceived the study, contributed to the collection of the data (was responsible for the collection of the pathology review data) and contributed to the data analysis and writing of the manuscript. JLH established the ABCFR and its data collection, conceived the study design, data analysis and interpretation and the writing of the manuscript. EM and DFS conducted the LASSO statistical analyses and contributed to the writing of the manuscript. GGG helped establish the ABCFR and its data collection and contributed to the writing of the manuscript. All authors read and approved the final manuscript.

## References

[B1] Pharoah PD Day NE Duffy S Easton DF Ponder BA Family history and the risk of breast cancer: a systematic review and meta-analysis Int J Cancer 1997 14 800 809 10.1002/(SICI)1097-0215(19970529)71:5<800::AID-IJC18>3.0.CO;2-B 9180149

[B2] Collaborative Group on Hormonal Factors in Breast Cancer Familial breast cancer: collaborative reanalysis of individual data from 52 epidemiological studies including 58,209 women with breast cancer and 101,986 women without the disease Lancet 2001 14 1389 1399 11705483 10.1016/S0140-6736(01)06524-2

[B3] Dite GS Jenkins MA Southey MC Hocking JS Giles GG McCredie MR Venter DJ Hopper JL Familial risks, early-onset breast cancer, and BRCA1 and BRCA2 germline mutations J Natl Cancer Inst 2003 14 448 457 10.1093/jnci/95.6.448 12644538

[B4] Loman N Bladström A Johannsson O Borg A Olsson H Cancer incidence in relatives of a population-based set of cases of early-onset breast cancer with a known *BRCA1 *and *BRCA2 *mutation status Breast Cancer Res 2003 14 R175 R186 10.1186/bcr632 14580253 PMC314401

[B5] Lee JS John EM McGuire V Felberg A Ostrow KL DiCioccio RA Li FP Miron A West DW Whittemore AS Breast and ovarian cancer in relatives of cancer patients, with and without BRCA mutations Cancer Epidemiol Biomarkers Prev 2006 14 359 363 10.1158/1055-9965.EPI-05-0687 16492929

[B6] Dite GS Whittemore AS Knight JA John EM Milne RL Andrulis IL Southey MC McCredie MR Giles GG Miron A Phipps AI West DW Hopper JL Increased cancer risks for relatives of very early-onset breast cancer cases with and without *BRCA1 *and *BRCA2 *mutations Br J Cancer 2010 14 1103 1108 10.1038/sj.bjc.6605876 20877337 PMC2965877

[B7] Swift M Reitnauer PJ Morrell D Chase CL Breast and other cancers in families with ataxia-telangiectasia N Engl J Med 1987 14 1289 1294 10.1056/NEJM198705213162101 3574400

[B8] Li FP Fraumeni JF Jr Soft-tissue sarcomas, breast cancer, and other neoplasms. A familial syndrome? Ann Intern Med 1969 14 747 752 5360287 10.7326/0003-4819-71-4-747

[B9] Lalloo F Varley J Moran A Ellis D O'Dair L Pharoah P Antoniou A Hartley R Shenton A Seal S Bulman B Howell A Evans DG *BRCA1, BRCA2 *and *TP53 *mutations in very early-onset breast cancer with associated risks to relatives Eur J Cancer 2006 14 1143 1150 10.1016/j.ejca.2005.11.032 16644204

[B10] Mouchawar J Korch C Byers T Pitts TM Li E McCredie MR Giles GG Hopper JL Southey MC Population-based estimate of the contribution of TP53 mutations to subgroups of early-onset breast cancer: Australian Breast Cancer Family Study Cancer Res 2010 14 4795 4800 10.1158/0008-5472.CAN-09-0851 20501846 PMC3228832

[B11] Turnbull C Ahmed S Morrison J Pernet D Renwick A Maranian M Seal S Ghoussaini M Hines S Healey CS Hughes D Warren-Perry M Tapper W Eccles D Evans DG Breast Cancer Susceptibility Collaboration (UK) Hooning M Schutte M van den Ouweland A Houlston R Ross G Langford C Pharoah PD Stratton MR Dunning AM Rahman N Easton DF Genome-wide association study identifies five new breast cancer susceptibility loci Nat Genet 2010 14 504 507 10.1038/ng.586 20453838 PMC3632836

[B12] Southey MC Ramus SJ Dowty JG Smith LD Tesoriero AA Wong EE Dite GS Jenkins MA Byrnes GB Winship I Phillips KA Giles GG Hopper JL Morphological predictors of *BRCA1 *germline mutations in young women with breast cancer Br J Cancer 2011 14 903 909 10.1038/bjc.2011.41 21343941 PMC3065278

[B13] Wong EM Southey MC Fox SB Brown MA Dowty JG Jenkins MA Giles GG Hopper JL Dobrovic A Constitutional methylation of the *BRCA1 *promoter is specifically associated with *BRCA1 *mutation-associated pathology in early-onset breast cancer Cancer Prev Res 2011 14 23 33 10.1158/1940-6207.CAPR-10-0212 PMC4030007 20978112

[B14] Hopper JL Giles GG McCredie MRE Boyle P Background, rationale and protocol for a case-control-family study of breast cancer Breast 1994 14 79 86 10.1016/0960-9776(94)90003-5

[B15] McCredie MRE Dite GS Giles GG Hopper JL Breast cancer in Australian women under the age of 40. Cancer Causes Control 1998 14 189 198 10.1023/A:1008886328352 9578296

[B16] Hopper JL Chenevix-Trench G Jolley D Dite GS Jenkins MA Venter DJ McCredie MR Giles GG Design and analysis issues in a population-based, case-control-family study of the genetic epidemiology of breast cancer and the Co-operative Family Registry for Breast Cancer Studies (CFRBCS) J Natl Cancer Inst Monogr 1999 14 95 100 10854492 10.1093/oxfordjournals.jncimonographs.a024232

[B17] Armes JE Egan AJ Southey MC Dite GS McCredie MR Giles GG Hopper JL Venter DJ The histologic phenotypes of breast carcinoma occurring before age 40 years in women with and without *BRCA1 *or *BRCA2 *germline mutations: a population-based study Cancer 1998 14 2335 2345 10.1002/(SICI)1097-0142(19981201)83:11<2335::AID-CNCR13>3.0.CO;2-N 9840533

[B18] Longacre TA Ennis M Quenneville LA Bane AL Bleiweiss IJ Carter BA Catelano E Hendrickson MR Hibshoosh H Layfield LJ Memeo L Wu H O'Malley FP Interobserver agreement and reproducibility in classification of invasive breast carcinoma: an NCI breast cancer family registry study Mod Pathol 2006 14 195 207 10.1038/modpathol.3800496 16341153

[B19] Page DL Anderson TJ Sakamoto G Page DL, Anderson TJ Infiltrating carcinoma: major histological types Diagnostic Histopathology of the Breast 1987 Edinburgh: Churchill Livingstone 193 235

[B20] Armes JE Trute L White D Southey MC Hammet F Tesoriero A Hutchins AM Dite GS McCredie MR Giles GG Hopper JL Venter DJ Distinct molecular pathogenesis of early-onset breast cancers in *BRCA1 *and *BRCA2 *mutation carriers: a population-based study Cancer Res 1999 14 2011 2017 10213514

[B21] McCredie MRE Dite GS Southey MC Venter DJ Giles GG Hopper JL Risk factors for breast cancer by oestrogen receptor and progesterone receptor status Br J Cancer 2003 14 1661 1663 10.1038/sj.bjc.6601293 14583766 PMC2394423

[B22] Tavtigian SV Oefner PJ Babikyan D Hartmann A Healey S Le Calvez-Kelm F Lesueur F Byrnes GB Chuang SC Forey N Feuchtinger C Gioia L Hall J Hashibe M Herte B McKay-Chopin S Thomas A Vallée MP Voegele C Webb PM Whiteman DC Australian Cancer Study; Breast Cancer Family Registries (BCFR); Kathleen Cuningham Foundation Consortium for Research into Familial Aspects of Breast Cancer (kConFab) Sangrajrang S Hopper JL Southey MC Andrulis IL John EM Chenevix-Trench G Rare, evolutionarily unlikely missense substitutions in *ATM *confer increased risk of breast cancer Am J Hum Genet 2009 14 427 446 10.1016/j.ajhg.2009.08.018 19781682 PMC2756555

[B23] Goldgar DE Healey S Dowty JG Da Silva L Chen X Spurdle AB Terry MB Daly MJ Buys SM Southey MC Andrulis I John EM BCFR; kConFab Khanna KK Hopper JL Oefner PJ Lakhani S Chenevix-Trench G Rare variants in the *ATM *gene and risk of breast cancer Breast Cancer Res 2011 14 R73 10.1186/bcr2919 21787400 PMC3236337

[B24] Chenevix-Trench G Spurdle AB Gatei M Kelly H Marsh A Chen X Donn K Cummings M Nyholt D Jenkins MA Scott C Pupo GM Dörk T Bendix R Kirk J Tucker K McCredie MR Hopper JL Sambrook J Mann GJ Khanna KK Dominant negative ATM mutations in breast cancer families J Natl Cancer Inst 2002 14 205 215 Erratum in. *J Natl Cancer Inst*, 2002, **94:**952 10.1093/jnci/94.3.205 11830610

[B25] Bernstein JL Teraoka S Southey MC Jenkins MA Andrulis IL Knight JA John EM Lapinski R Wolitzer AL Whittemore AS West D Seminara D Olson ER Spurdle AB Chenevix-Trench G Giles GG Hopper JL Concannon P Population-based estimates of breast cancer risks associated with *ATM *gene variants c.7271T>G and c.1066-6T>G (IVS10-6T>G) from the Breast Cancer Family Registry Hum Mutat 2006 14 1122 1128 10.1002/humu.20415 16958054

[B26] Breslow NE Day NE Statistical Methods in Cancer Research. Volume II - The Design and Analysis of Cohort Studies 1987 Lyon, France: International Agency for Research on Cancer 3329634

[B27] Park T Casella G The Bayesian lasso J Amer Statistical Assoc 2008 14 681 686 10.1198/016214508000000337

[B28] Kyung M Gill J Ghosh M Casella G Penalized regression, standard errors, and Bayesian lassos Bayesian Analysis 2010 14 369 412 10.1214/10-BA607

[B29] StataCorp Stata Statistical Software: Release 11.0 2010 College Station, TX: StataCorp LP

[B30] The MathWorks, Inc. MATLAB version 7.13.0 2011 Natick, Massachusetts: The MathWorks Inc

[B31] Lakhani SR Jacquemier J Sloane JP Gusterson BA Anderson TJ van de Vijver MJ Farid LM Venter D Antoniou A Storfer-Isser A Smyth E Steel CM Haites N Scott RJ Goldgar D Neuhausen S Daly PA Ormiston W McManus R Scherneck S Ponder BA Ford D Peto J Stoppa-Lyonnet D Bignon YJ Struewing JP Spurr NK Bishop DT Klijn JG Devilee P Multifactorial analysis of differences between sporadic breast cancers and cancers involving *BRCA1 *and *BRCA2 *mutations J Natl Cancer Inst 1998 14 1138 1145 10.1093/jnci/90.15.1138 9701363

[B32] Lakhani SR van de Vijver MJ Jacquemier J Anderson TJ Osin PP McGuffog L Easton DF The pathology of familial breast cancer: predictive value of immunohistochemical markers estrogen receptor, progesterone receptor, HER-2, and P53 in patients with mutations in *BRCA1 *and *BRCA2* J Clin Oncol 2002 14 2310 2318 10.1200/JCO.2002.09.023 11981002

[B33] Jiang X Castelao JE Chavez-Uribe E Fernandez Rodriguez B Celeiro Muñoz C Redondo CM Peña Fernandez M Novo Dominguez A Pereira CD Martínez ME García-Caballero T Fraga Rodriguez M Antúnez J Carracedo A Forteza-Vila J Gago-Dominguez M Family history and breast cancer hormone receptor status in a Spanish cohort PLoS One 2012 14 e29459 10.1371/journal.pone.0029459 22238615 PMC3253097

[B34] Lorenzo BJ Hemminki K Familial association of histology specific breast cancers with cancers at other sites Int J Cancer 2004 14 430 435 10.1002/ijc.11713 14961583

[B35] Tutera AM Sellers TA Potter JD Drinkard CR Wiesner GL Folsom AR Association between family history of cancer and breast cancer defined by estrogen and progesterone receptor status Genet Epidemiol 1996 14 207 221 10.1002/(SICI)1098-2272(1996)13:2<207::AID-GEPI6>3.0.CO;2-4 8722747

[B36] Ellberg C Olsson H Breast cancer patients with lobular cancer more commonly have a father than a mother diagnosed with cancer BMC Cancer 2011 14 497 10.1186/1471-2407-11-497 22117567 PMC3241222

[B37] Martins FC De S Almendro V Gönen M Park SY Blum JL Herlihy W Ethington G Schnitt SJ Tung N Garber JE Fetten K Michor F Polyak K Evolutionary pathways in BRCA1-associated breast tumors Cancer Discov 2012 14 503 511 10.1158/2159-8290.CD-11-0325 22628410 PMC3738298

[B38] Masciari S Dillon DA Rath M Robson M Weitzel JN Balmana J Gruber SB Ford JM Euhus D Lebensohn A Telli M Pochebit SM Lypas G Garber JE Breast cancer phenotype in women with TP53 germline mutations: a Li-Fraumeni syndrome consortium effort Breast Cancer Res Treat 2012 14 1125 1130 10.1007/s10549-012-1993-9 22392042 PMC3709568

[B39] Flanagan JM Cocciardi S Waddell N Johnstone CN Marsh A Henderson S Simpson P da Silva L kConFab Investigators Khanna K Lakhani S Boshoff C Chenevix-Trench G DNA methylome of familial breast cancer identifies distinct profiles defined by mutation status Am J Hum Genet 2010 14 420 433 10.1016/j.ajhg.2010.02.008 20206335 PMC2833389

